# Improving the sustainability and effectiveness of photovoltaic evaporative cooling greenhouse in the Sahel

**DOI:** 10.1038/s41598-024-51352-9

**Published:** 2024-05-16

**Authors:** Alio Sanda M. Djibrilla, Adamou Rabani, Karimoun M. Illyassou, Atto H. Abdoulkader, Drame Yaye Aissetou

**Affiliations:** 1 WASCAL Doctorate Research Program-Climate Change and Energy, Faculty of Science and Technique, Abdou Moumouni University, Niamey, Niger; 2 Department of Physics, Faculty of Science and Technique, Abdou Moumouni University, Niamey, Niger; 3 Department of Agriculture Engineering and Forestry, Faculty of Agronomy, Abdou Moumouni University, Niamey, Niger; 4 Department of Research and Development, DRAMS, Niamey, Niger; 5 Enterprise in Research and Development, Sahel Agro, Niamey, Niger; 6 Department of Chemistry, Ecole Normale Supérieure, Abdou Moumouni University, Niamey, Niger

**Keywords:** Environmental sciences, Energy science and technology, Engineering

## Abstract

Anthropogenic climate change has caused worldwide extreme weather events including droughts, floods and heatwaves. It disproportionately affects developing countries through food insecurity. Greenhouse is important and relevant to the food-energy-water security in many regions. This study investigates the thermal behavior of photovoltaic evaporative cooling greenhouse made with eco-friendly coolers. The cooling potential of local plant materials was assessed under ambient conditions. Experimental thermal data obtained from optimized evaporative cooling system equipped with *Hyphaene thebaica* fibers (HF-pad) and conventional Celdek pad (C-pad), were used in heat and mass transfer equations to derive the greenhouse cooling performances. Computational fluid dynamics analysis software was used to investigate the refrigerant fluid distribution in the greenhouse. Cooler using HF-pad allows to keep the microclimate below 25 °C, with maximum moisture rate up to 80%, under harsh ambient conditions (temperature: 30–45 °C, humidity: 10–15%). HF-pad had the highest cooling coefficient of performance (COP = 9 against 6 for C-pad), the best cost to efficiency ratio (CER = 5; 4 times less than C-pad) and the lowest outlet temperature (20.0 °C). Due to higher outlet air velocity (1.116 m/s against 0.825 m/s for HF-pad), C-pad cooler spread cool air (20.5 °C) up to 1.25 m farther than its counterpart, creating higher pressure in the atmosphere (1.42 Pa against 0.71 Pa), with 2 times turbulent kinetic energy (0.014 J/kg). HF-pad presented cooling performances that compete with conventional pads. Moreover, optimization of HF-pad frame engineering and the technology scaling up to industrial level can allow better thermal and economic performances.

## Introduction

Anthropogenic climate change is a change in weather patterns caused by humans’ day-to-day activities. The resulted global warming has led to sea level rise and more occurring weather extremes such as droughts, floods and heat waves^1^. Climate change threatens the world both across the lands and over sea ecosystems, affecting disproportionately developing countries and their inhabitants with food insecurity and extreme weather events. Moreover, it causes new diseases vectors proliferation and slowing down socioeconomic development in the global south^2^ where most vulnerable people, middle to low-income earners, are found. They survive through farming activities such that livestock, market gardening and poultry to meet their daily needs^3^. Approaches including improved and adapted seeds^4^ as well as mitigated agriculture technologies (solar pumping, drip irrigation and agri photovoltaic) have been proposed as key adaptation solutions to climate variability and change. The accessibility and replicability of the proposed solutions often create mistrusts between local farmers and solution providers. Therefore, agriculture technology like evaporative cooling greenhouse, a controlled microclimate, made from local biomass materials through an inclusive approach could help vulnerable farmers to keep productivity high while using traditional and improved crop varieties. Greenhouse is a close structure in which crop cultivation can be done under conducive climate conditions, allowing optimum crop growth and yield, and better protection against invaders^5,6^. Thus, greenhouse technology is one of the best ways to reduce climate risks and losses in agricultural productivity^7–11^. Under Sahelian dry and hot weather conditions, cooling is necessary in order to keep conducive microclimate^12^. Many cooling methods have been used from natural convective cooling through the use of temperature gradient^5^, roof cooling technics^5^, photo selective covering materials^6,7^, fog systems^12^, to heat exchangers cooling methods^8^ and even combined cooling methods^5,8,9^. However, the most cost effective cooling technology adapted for the Sahel and accessible to middle to low income people, especially farmers, still being eco-friendly, are evaporative coolers^10,11^. Advances in evaporative cooling showed that these technologies could provide indoor comfort under tropical climate conditions^6^. Cooling a greenhouse with evaporative pads, was reported to considerably lower internal air temperature and decrease water vapor pressure (VPD)^11^. The microclimate of the greenhouse was successfully maintained below 28 °C and 80% relative humidity with a combined forced ventilation fueled by solar photovoltaic energy system^11,12^. This microclimate could be channeled for optimum plant growth, livestock breeding or others similar uses by providing adequate cool and humid air while not emitting greenhouse gases responsible for global warming and climate change^10,13–15^. Elsewhere, knowing the cooling fluid flow pattern and thermal distribution within the greenhouse help optimize the greenhouse cooling system operation^16^. Computational Fluid Dynamics (CFD) is a tool that can numerically investigate these flow parameters distribution^15–19^. CFD is a proven investigation tool widely used to understand better the airflow and nutrient like artificial CO_2_ distribution inside greenhouse. The application of CFD models allowed to acquire the appropriate thermal and solar radiation distribution within Photovoltaic (PV) mounted greenhouse^17^. Moreover, CFD analysis allowed to assess the cooling fluid flow, temperature variation and homogeneity (kinetic energy turbulence) for a better ventilation in the greenhouse^16,17^. In recent years, CFD analysis is widely used to evaluate the influence of the greenhouse key components (soil, crops, wall, roof…) interactions on the indoor thermal parameters^16,18,19^. In this work, the fluid flow characteristics within a photovoltaic evaporative cooling greenhouse (PV-ECG) adapted for the Sahel was investigated using coolers technology equipped with locally made *Hyphaene thebaica* fibers pad against commercial Celdek pad. The alternative wet-pad material (*Hyphaene thebaica*) was chosen after a deep evaluation process of the cooling performances of many local plant materials, traditionally used by farmers for cooling needs^10,13^. Field climatic parameters recorded in situ and thermophysical data obtained at the laboratory prototype level and from the installed PV-ECG were used to assess the alternative HF-pad cooling potential against conventional C-pad. The greenhouse key cooling performances (cooling capacity, coefficient of performance and cost to efficiency ratio) were derived from established energy and mass balance thermodynamic equations^15,16^. In order to have a global understanding of the refrigerant fluid distribution in the whole greenhouse, ANSYS CFD analysis software was used to simulate the cooling fluid flow velocity, temperature, density and turbulent kinetic energy distribution patterns. To avoid conventional costly and unsustainable cooling system in the Sahel with air conditioner connected to the grid or through generators fueled by carbonated fossil fuel, a standalone photovoltaic system was used to power the evaporative cooling system (water pumping and distribution and ventilators).

## Materials and methods

### Experimental setup

The site is located in the department of Say (13°10.1969′N and 002°19.0080′E), 40 km from Niamey (Niger). The built greenhouse covered an area of 50 m^2^ (span = north–south, length = 10 m, width = 5 m, height = 3.66 m and roof tilt angle = 15°). This study was done using in situ climatic and thermophysical data collected from an experimental evaporative cooling greenhouse powered by a standalone photovoltaic system. The cooling system is powered by six (6) solar photovoltaic modules (each 260 W) installed on the greenhouse roof. An inverter of 5 kVA and 4 batteries of 150 Ah each for energy storage were added for a smooth operation of the cooling system. The greenhouse prototype was designed using Robot Structural Analysis PRO 2017, a structural load analysis software (including potential wind load simulation) in order to have a more resilient constructible model. Sketch Up 3D software helped to get the greenhouse 3D structure for a complete design to technically and efficiently place the key components (cooling, water pumping and distribution and power systems) by doing all needed adjustments before field installation. The greenhouse was equipped with the desired cooling pads (alternative and conventional), water pumping and draining system. The cooling was based on evaporative cooling pad (H × W × ε = 0.5 × 0.5 × 0.04 m) locally made using alternative cellulosic materials (here HF-pad) against commercial Celdek-pad. The pads were inserted along the greenhouse sides, ventilators (1per pad) installed at the windy sidewall of the greenhouse, drawing in additional air through the wet-pad. The water distribution system installed across the pad top cross section is a close recyclable system which regularly provides water to the cooling pad. Outdoor of the greenhouse and the internal microclimate were equipped with calibrated devices allowing collecting in situ the key climatic and environmental parameters. Soil temperature and pH were measured with a digital pH/temperature soil analyzer (KETOTEK). Leaf temperature was measured using a contactless infrared thermometer (KETOTEK). The relative humidity and air temperature were measured using a thermo-hygrometer (Digital, Temperature from − 20 to 60 °C, Humidity from 5 to 95%). The intensity of the light is taken through the electronic luxmeter (Digital Luxmeter/photometer TASI-8721). The performance of the different biomaterial pads and conventional Celdek-pad were preliminary assessed at the laboratory level with thermal investigation set-up; a thermodynamically isolated duct prototype made of 2 aluminum sheets filled with glass wool (thickness 4 cm). It is composed of two modules connected by the pad holder which allows controlled air (velocity, temperature, relative humidity, specific heat capacity) to pass through the inserted wet-pad to simulate field conditions^15,16^. Simulation data were collected remotely with sensors via a computer from both the inlet and outlet environments^10^. This process was repeated for the alternative HF-pad and commercial C-pad to compare measured thermal data and calculated performances. The built evaporative cooling greenhouse was also equipped with similar sensors in order to collect the field data and compare them with laboratory findings. During the experiment, the input air (ambient) average temperature and relative humidity were respectively between 30–45 °C and 10–15%, characteristics of hot and dry season (March–June), unbearable for seasonal horticulture. Table 1 presents the characteristics of the air passing the cooling pads.

**Table 1:** Thermal characteristics of inlet air from the environment towards coolers^13^.

Parameters	Inlet to pad from outside environment	
	Hyphaene fibers	Celdek
Average temperature of incoming dry air, T_in_ (°C)	35.67	34.58
Average relative humidity of incoming dry air, RH_in_ (%)	13.83	13.50

### Cooling pad characteristics

The reference Celdek cooling pad (C-pad) is a proven conventional pad designed to provide maximum cooling and low pressure-drop in controlled environment. It is impregnated and treated in order to improve the made cellulosic absorbance and lifetime while assuring uniform air flow and higher evaporative cooling efficiency^14^. The alternative HF-Pad fibers were extracted from local *Hyphaene thebaica* plants, dried at ambient temperature and filled in the pad frame. Table 2 presents the physical characteristics of HF-pad and reference C-pad.

**Table 2:** Physical parameters of Hyphaene fiber pad against commercial Celdek pad^13^.

Pad type	Flute size (mm)	Structure	Arrangement	Texture
Hyphaene fiber	1	Coil	Disordered: packing to filling	
Celdek	1.5	Honeycomb	Ordered: 1 cm between crests	

The cooling and economic potentials of the conventional C-pad and the alternative cooling HF-pad were characterized. The saturation efficiency (eff), cooling capacity (q), coefficient of performance (COP) and the cost to efficiency ratio (CER) were derived using the following thermodynamic Eqs. (1), (2), (3), (4), (5), (6) and (7). In order to assess the alternative pad cooling potential, the amount of moisture (saturation efficiency) was firstly evaluated, then the amount of heat (cooling capacity) that the process can allow to remove from the controlled environment was determined. In a well homogenized microclimate, the cooling capacity of the system is equal to the change in the refrigerant (air) specific enthalpy. The saturation efficiency and the cooling capacity of the pad were given by Eqs. (1) and (2)^10^:

1 $${\text{Saturation}}\;{\text{efficiency}}\;{\text{is}}\;{\text{given}}\;{\text{by}}:{\text{ eff}} = \frac{{{\text{T}}_{{{\text{in}}}} { } - {\text{ T}}_{{{\text{out}}}} }}{{{\text{T}}_{{{\text{in}}}} { } - {\text{ T}}_{{{\text{wb}}}} }}$$

2 $${\text{Cooling}}\;{\text{capacity}}\;{\text{is}}\;{\text{given}}\;{\text{by}}:\;{\text{q}}\; = \;{\text{ma Cpa}}\left( {{\text{T}}_{{{\text{in}}}} - {\text{T}}_{{{\text{out}}}} } \right)$$

where T_in_ (K) and T_out_ (K) are the inlet and outlet temperatures respectively, T_wb_ (K) is the wet bulb temperature, q is the cooling capacity (J s ^−1^), m_a_ and C_pa_ are the mass flow rate (kg s^−1^) and specific heat capacity of air (J kg^−1^ K^−1^).

The greenhouse outdoor environment and indoor microclimate heat and mass transfer investigation can allow the determination of the system cooling coefficient of performance. When the thermal equilibrium was reached between outdoor and indoor, the following energy balance equation could be written^10^:

3 $${\text{maha}}_{{1}} + {\text{mv}}_{{1}} {\text{hv}}_{{1}} + {\text{mv}}_{{1}} {\text{hw}} = {\text{maha}}_{{2}} + {\text{mv}}_{{2}} {\text{hv}}_{{2}} + {\text{mv}}_{{2}} {\text{hw}} + {\text{q}}$$

With ha, hv and hw being the enthalpy of air, of water vapor and of water respectively. mv represent the mass of water vapor in the air stream. The indices 1 and 2 represent inlet and outlet parameters.

Using the heat loss q, the heat transfer coefficient could be derived^10^:

4 $${\text{Heat}}\;{\text{loss}}:{\text{q}} = {\text{h}}_{{\text{H}}} {\text{A}}_{{\text{s}}} \Delta {\text{T}}$$

h_H_ heat transfer coefficient (Js^−1^ m^−2^ K^−1^), A_s_ is the surface area (m^2^) and ∆T = (∆T_1_ − ∆T_2_)/ln(∆T_1_/∆T_2_) is the logarithmic mean (average) of the temperature between the hot and cool fluid flows (outdoor and indoor), with the cooling pad as the boundary line^10^.

By analogy with heat transfer (energy transfer due to temperature gradient) between the two environments, the mass of water transfer rate (mol s^−1^) during the cooling process (energy transfer resulted from concentration difference) can be obtained. Similarly, the mass transfer rate (m_e_) is function of the mass transfer coefficient (h_M_), the effective mass transfer area (A_s_) and the driving force density difference (**∆**ρv)^10^:

5 $${\text{me}} = {\text{h}}_{{\text{M}}} {\text{A}}_{{\text{s}}} \Delta \rho {\text{v}}$$

where h_M_ (mol s^−1^ m^−2^)/(mol m^−3^) = m s^−1^) is the mass transfer coefficient and ∆ρv (mol m^−3^) is the log mean density.

In addition to the valorization of the locally available biomass, to increase the sustainability condition of the designed greenhouse, a standalone photovoltaic system was used to tap in the huge renewable energy potential in the region (7 kwh/m^2^/day, with 8–10 h sunshine daily)^20^, to power the water distribution pump and the ventilators. The cooling coefficient of performance (COP) which assesses the effectiveness of the greenhouse cooling system is the ratio of the heat removed from the greenhouse microclimate to the power from the solar PV source used by the whole cooling system^10^.

6 $${\text{Coefficient}}\;{\text{of}}\;{\text{Performance}}\;\left( {{\text{COP}}} \right)\; = \frac{{{\text{q}}_{{{\text{pad}}}} }}{{{\text{P}}_{{{\text{fan}}}} + {\text{P}}_{{{\text{pump}}}} }}$$

where q_pad_ is the cooling capacity rate of the pad (J/s), P_pump_ (18W) and P_fan_ (75W) are power of the pump used to bring the cooling water and the blower’s power respectively.

In addition to the technical performances of the evaporative cooling greenhouse, the system economic efficiency would play a role in the technology deployment. In the global south, the cost to efficiency ratio (CER) would be a key indicator to evaluate accessibility to innovation^10^.

7 $${\text{Cost}}\;{\text{to}}\;{\text{efficiency}}\;{\text{ratio}}\;\left( {{\text{CER}}} \right)\; = \frac{{{\text{Cost}}}}{{{\text{eff}}}}$$

### Computational fluid dynamic (CFD) analysis

**Figure 1:**
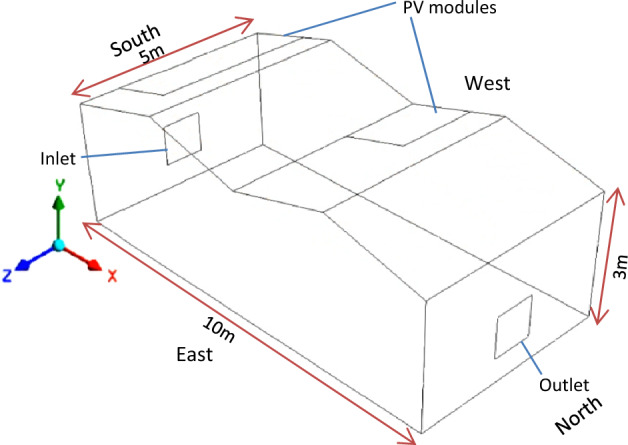
PV Greenhouse orientation.

CFD is a strong numerical tool which can help to understand the refrigerant flow and distribution in the greenhouse microclimate. It allows to capture the effect of the new cooling pad on the cooling air distribution direction and extent in the controlled environment. Therefore, the cooling greenhouse was carefully drawn in SpaceClaim 2022 respecting the field dimension before being transferred in Workbench for CFD analysis. Then, the air flow and distribution patterns through both HF-pad and commercial C-pad were studied using output thermal data and obtained performances. The flow inside the greenhouse (Fig. 1) was assumed 3D, steady state, incompressible ideal gas behavior and turbulent^21,22^.

#### Numerical model

ANSYS Fluent student version R2 2022 was used for the simulation. The greenhouse had a coverage area of 50 m^2^ with a total height of 3.66 m and a roof slope of 15°.

The configuration was pressure–velocity coupling with SIMPLEC algorithm leading to the formulation of mass conservation equation. The discretization of energy equation, turbulence kinetic energy, turbulence dissipation rate, special momentum and convective heat transfer equations were taken into account by a Second-Order Upwind (SOU) scheme, which materialized the Reynolds Averaged Navier–Stokes (RANS) transport equation^16,17,23^. This complex second order differential equation, with no analytical solution at our knowledge, is proven to be an interesting tool to develop models for fluid flow and distribution investigations^16–19,24^. In the used CFD software, the energy distribution within the greenhouse was based on RANS equation^16,24^:

8 $$\uprho \,{\text{U}}_{{\text{j}}} \frac{\partial \Phi }{{\partial {\text{x}}_{j} }} = \frac{\partial }{{\partial {\text{x}}_{j} { }}}\left( {\Gamma \frac{\partial \Phi }{{\partial {\text{x}}_{j} { }}}} \right) + {\text{S}}^{\Phi }$$

where Φ stands for the three velocity components U, V, W, the temperature T (K), the spectral intensity I, the kinetic energy of turbulence k, the dissipation rate ε, the specific humidity w (kg _water_ /kg _moist air_) and pressure P, and the parameters Γ and S^Φ^ represent the diffusion coefficient and source of term of Φ.

#### Boundary conditions

As boundary conditions, all transport properties (viscosity, thermal conductivity, diffusivity)^10,13^ were taken as resulted from the model and the inlet temperature, the minimum temperature from the coolers (20.00 °C for HF pad and 20.50 °C for Celdek pad). All side walls are assumed semitransparent and adiabatic with thickness 4 mm, except solar panels which were assumed opaque with thickness 40 mm. Mixed thermal conditions (radiative and convective heat transfer) were applied to all walls and solar modules.

## Results and discussion

### Comparative thermodynamic properties of Hyphaene fibers and Celdek pads

The average thermophysical values and the cooling performances obtained for *Hyphaene* fibers pad and Celdek pad are presented in Table 2. The ambient air average temperature and relative humidity recording during the study period were respectively 35 °C and 14%. The air temperature substantially dropped (− 14 °C) when the outdoor hot air passed through the alternative Wet-pad (HF-pad). The greenhouse indoor average relative humidity increased more than two times with HF-pad and reached 33%, against 50% for C-pad. The cooling saturation efficiency (eff) value obtained with HF-pad could allow the greenhouse microclimate to reach a maximum amount of moisture (77%), against 79% for Celdek-pad. The observed relative humidity difference can result from the ordered-structure and the industrial frame design of the C-Pad compared to the raw HF-fibers used as usual by farmers for their cooling needs. The HF-pad has huge potentials which can make a great difference with available commercial pads. It has the coolest output air (20 °C) against the conventional Celdek-pad (20.5 °C). With a limited mass transfer coefficient (hM = 0.9 kg/s) for similar heat transfer coefficient with Celdek pad (4.5 kW/m^2^ ^c^C), HF-pad will prevent invader’s introduction in the greenhouse when playing adequately its cooling function. According to the obtained cooling performances derived values, HF-pad can be a valuable alternative under harsh Sahelian climatic conditions. The greenhouse equipped with HF-pad presents better cooling coefficient of performance (COP = 9) compared to conventional C-pad (COP = 6), and has the lowest cost to efficiency ratio, CER HF-pad = 5.25; which means 4.5 times less expensive compared to commercial C-pad (CER = 20.73) (USD currency rate by the 30th of June 2023). The thermodynamic properties investigation conducted on the experimental cooling greenhouse has confirmed the preliminary laboratory thermophysical investigation data and derived cooling performances obtained with the laboratory evaporative cooling prototype^10,13^. Thermodynamic properties of the studied pads are presented in Table 3.The adequate thermophysical parameter values and highly cooling competitive performance of HF-pad against conventional C-pad, its large availability and cost-effectiveness can make HF-pad technology more appropriate for the region.

**Table 3:** Thermodynamic parameters of studied pads^13^.

Parameters	Cooling pads	
	Hyphaene fibers	Celdek
Minimum temperature from the coolers, T_out min_ (°C)	20.00	20.50
Average temperature from the coolers, T_out_ (°C)	21.83	20.92
Average wet bulb temperature, T_wb_ (°C)	17.83	17.42
Average relative humidity of wind from coolers, RH_out_ (%)	33.33%	50.00%
Moist air velocity from coolers, v_2_ (m/s)	0.83	1.17
Average saturation efficiency, eff (%)	77.57	79.60
Average mass flow rate, ma (kg/s)	0.012	0.012
Average cooling capacity, q (kJ/s)	0.17	0.17
Average heat transfer coefficient, h_H_ (kW/m^2^ ^c^C)	4.70	4.52
Average Mass transfer coefficient, h_M_ (kg/s)	0.88	1.40
Average Coefficient Of Performance, COP	9.04	6.56
Cost to Efficiency Ratio, CER	5.25	20.73

### CFD analysis over the greenhouse

The cooling refrigerant flow distribution in the greenhouse was investigated on different plans (horizontal and vertical) using CFD analysis software. For the alternative and conventional pads, a comparative study was conducted to understand the cooling fluid thermal behaviors considering air velocity, temperature, density, turbulent kinetic energy and dissipation rate. For all these thermal parameters, their distribution along the greenhouse horizontal and vertical plans were viewed respectively on plan ZX (Y = 0.75 m) and XY (Z = − 2.5 m). For each investigation, the referential origin was positioned at the pad middle, space coordinates (0, 0.75, 0) and (0, 0, − 2.5), respectively for the horizontal and vertical plans.

#### Greenhouse cooling air velocity distribution analysis with CFD software

**Figure 2:**
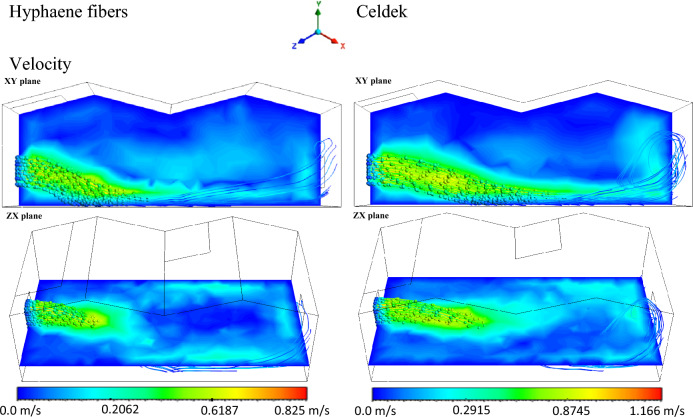
Velocity contours.

**Figure 3:**
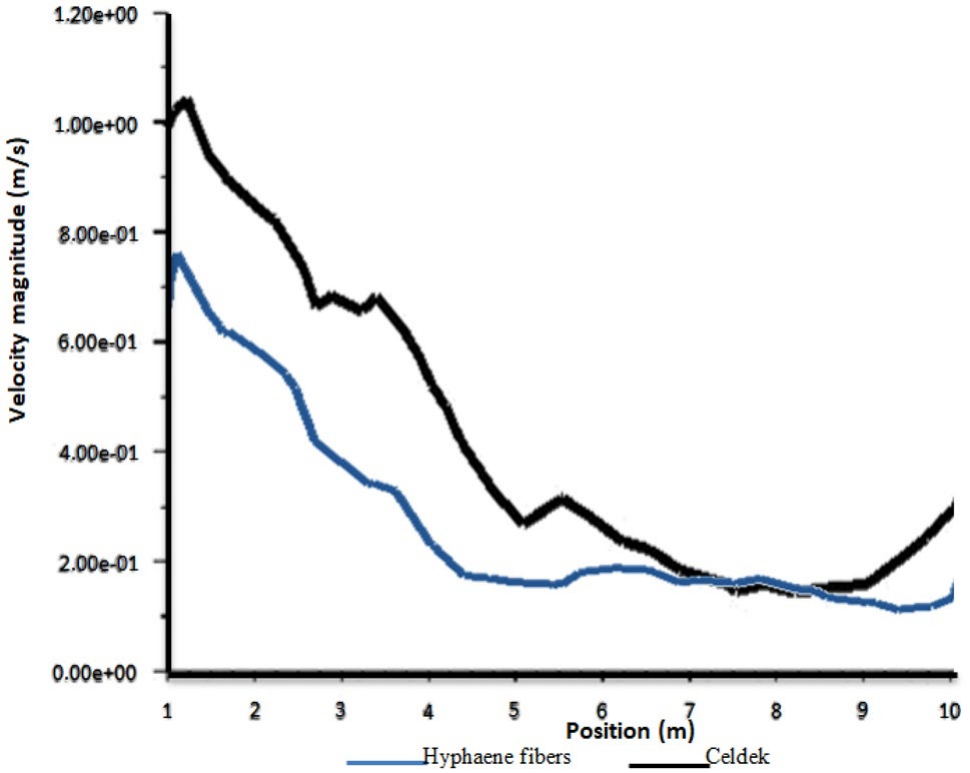
Plot of velocity magnitude from inlet to outlet.

Due to the difference in pad structure, the air velocity from the C-pad (1.166 m/s) and HF-pad (0.825 m/s) did not have the same distribution trend in the greenhouse (Fig. 2). The horizontal and vertical air spread by the conventional wet-pad is more important compared to alternative wet-pad. However, the C-pad air velocity magnitude dropped faster than that of the HF-pad. Indeed, as one moves away from the inlet, the two velocity magnitudes decrease similarly and equal at about 7.5 m from the inlet (Fig. 3). It was proven that air velocity affects many other key thermal parameters such as temperature, density and pressure in cooling greenhouse^8,25^. Thus, the observed horizontal and vertical air distribution trends will affect the greenhouse cooling system, impacting plants growth. Indeed, a low air flow rate was reported to be disadvantageous in a greenhouse of length around 12 m making cooling not efficient^26^. The low air velocity observed for HF-pad produces a weak internal air exchange rate causing air temperature rise and generate an insufficient relative humidity in the greenhouse, creating conditions not bearable for plant growth under harsh climatic areas^25^. Hence, additional ventilation is necessary to secure cool environment in the whole microclimate. It was also seen through the horizontal and vertical air distribution patterns that the HF-pad can be a promising alternative cooling pad. Similarly to C-pad, its frame improvement can contribute to increase the inlet air velocity for more cooling air distribution.

#### Greenhouse cooling air temperature distribution analysis with CFD software

**Figure 4:**
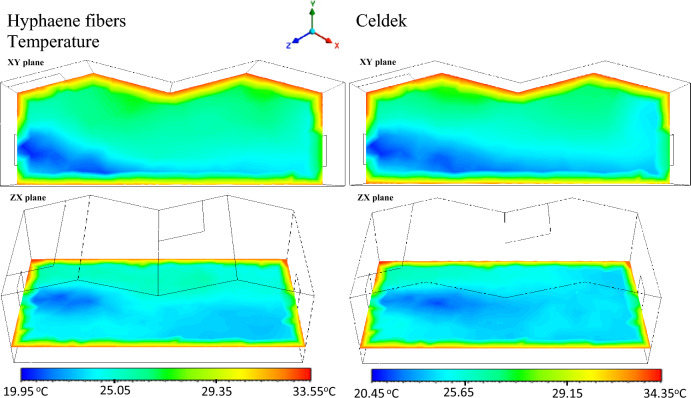
Temperature contours.

**Figure 5:**
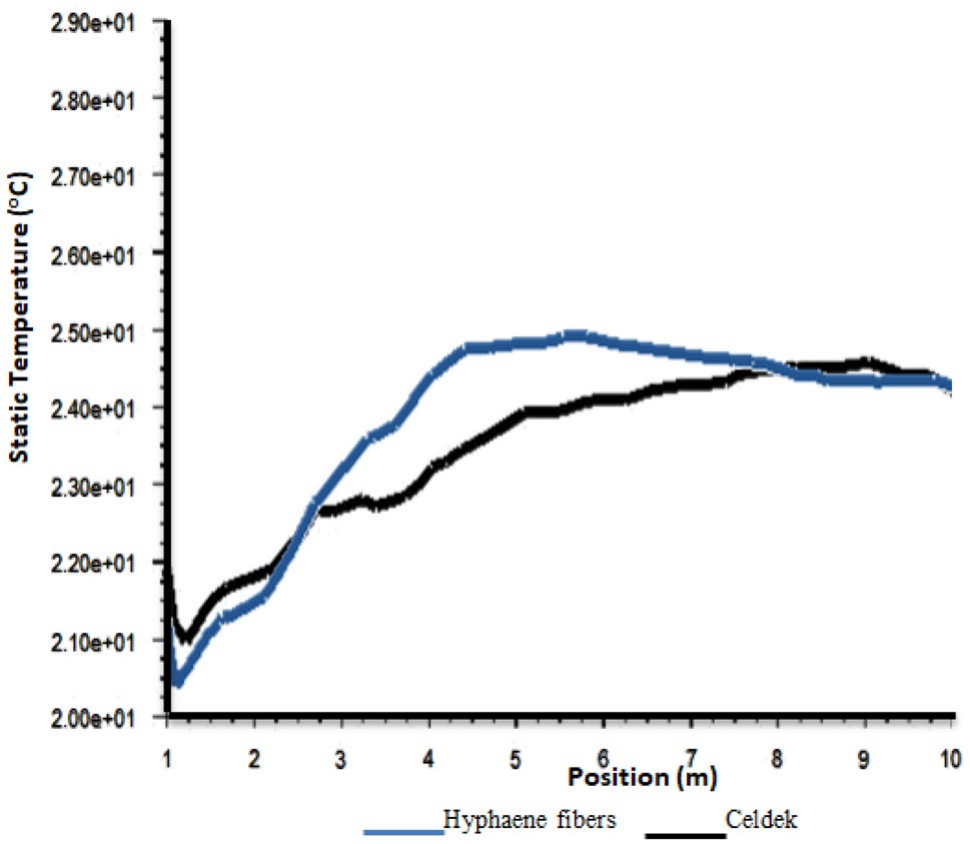
Plot of static temperature from inlet to outlet.

It was previously seen that wet-pad nature affects the cooling air distribution in the greenhouse. The observed microclimate temperature contours (Fig. 4), were in good agreement with the velocity of air stream previously observed in the greenhouse. Despite HF-pad relative weaker air velocity against commercial C-pad, it produced cooler microclimate up to 3 m (see statistic temperature vs distance plot). However, due to its low air velocity, above 3 m, the air temperature increased rapidly compared to commercial pad (see Fig. 5). For the two pads, along the inlet–outlet line, the air stream temperature was kept below 25 °C, a bearable temperature for most horticulture crops cultivated in the Sahel^27,28^. However, around the greenhouse roof vicinities and side extremities, temperature could reach 33.55 °C (Hyphaene fibers pad) and 34.55 °C (Celdek pad), creating a gradient of temperature of 6.35 and 6.95 °C respectively. Similar results were reported with heat accumulation increase up to 8 °C between the floor and the roof^29^. In fact, air temperature rose up from soil surface, crop canopy to the roof level, and was induced by buoyancy forces within the canopy^25^ even though the presence of vents could accelerate the rate of heat and water vapor losses to outside environment^25^.

#### Greenhouse cooling air density distribution analysis with CFD software

**Figure 6:**
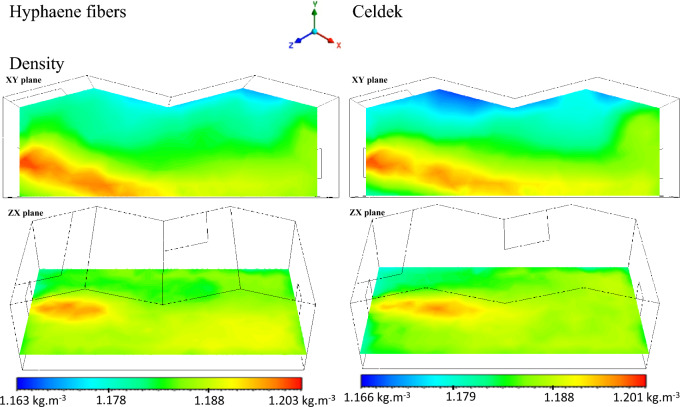
Density contours.

**Figure 7:**
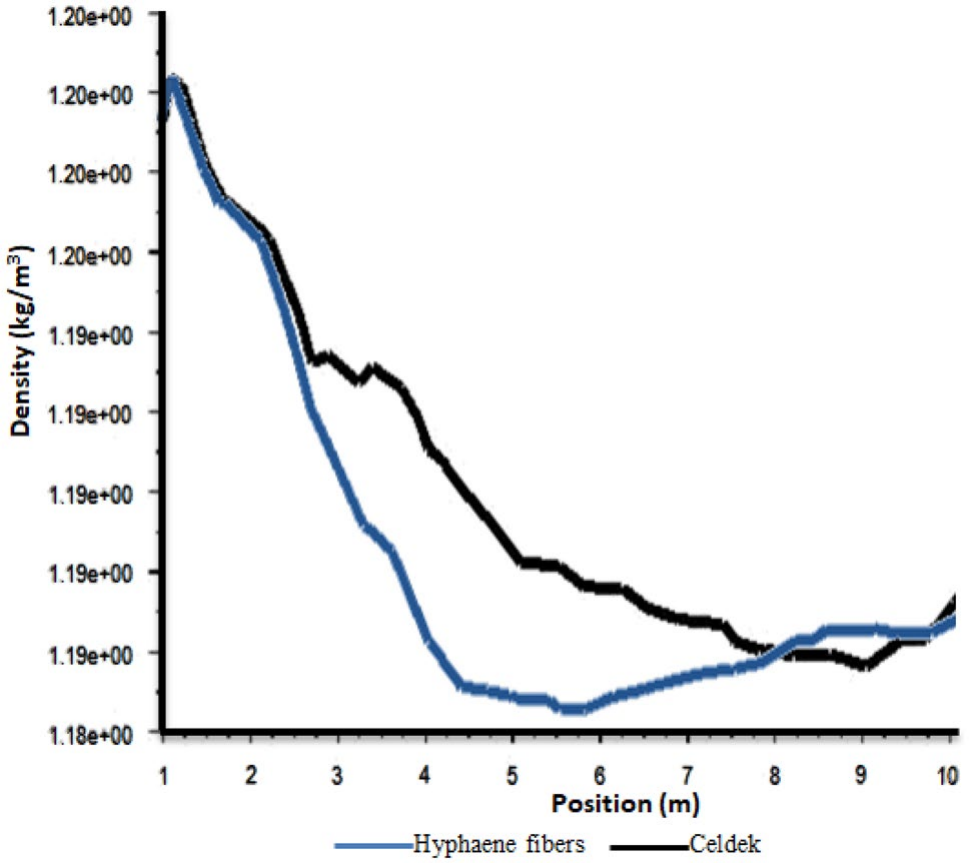
Plot of density from inlet to outlet.

**Figure 8:**
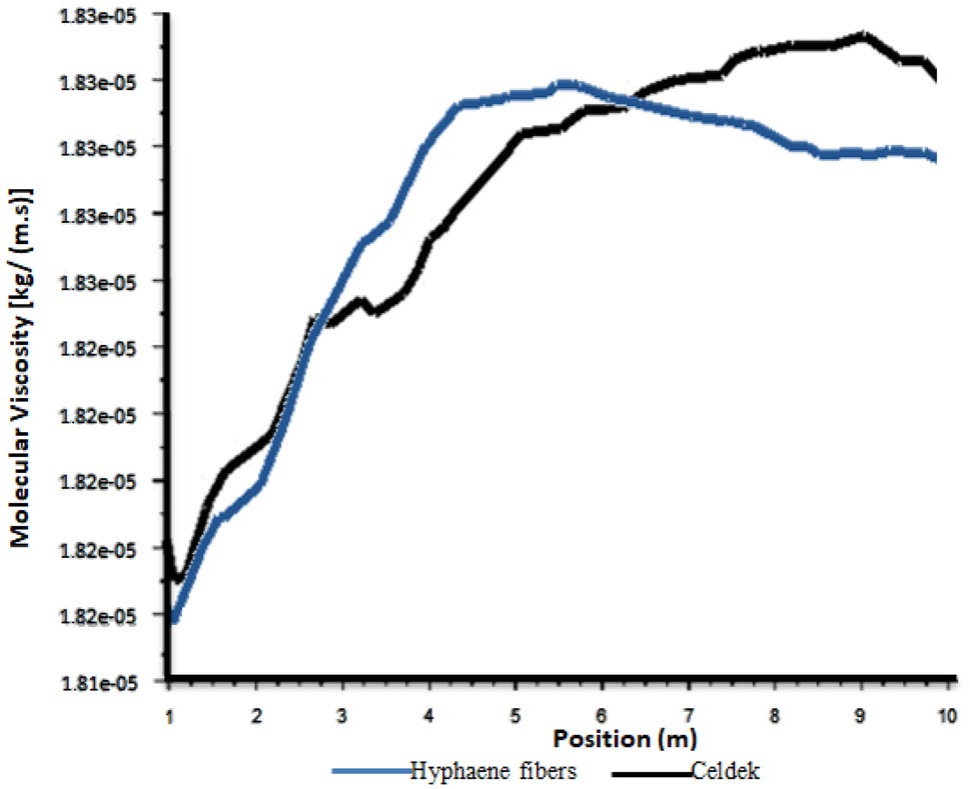
Plot of molecular viscosity from inlet to outlet.

Density was at its highest value (1.2 kg/m^3^) at the pad level and at its lowest value (1.16 kg/m^3^) at the roof (see Fig. 6). It was observed also similar air density values close to the two wet-pads confirming laboratory and field data which gave them the same air flow trend (0.012 kg/s). Despite the measured relative low air velocity observed for the alternative pad compared to conventional one, the same air density pattern was observed for the two pads between 0 to 3 m in the greenhouse (see Fig. 7). This can be explained by the fact that closer to the cooling wet-pad, HF-pad produced microclimate cooler than the C-pad. Indeed, cooler condition limits rapid air molecules expansion, having more important air density (Fig. 8). However, after 3 m, the air density observed for the alternative pad decreases strongly compared to the conventional Celdek pad. This can be linked to the important temperature increase after 3 m, when the greenhouse is equipped with alternative HF-pad compared to C-pad. The observed temperature enhancement can create warmer conditions allowing air molecules to move faster, producing rapid air expansion that can explains the important air density decrease between 3 and 6 m.

#### Greenhouse kinetic turbulent energy distribution analysis with CFD software

**Figure 9:**
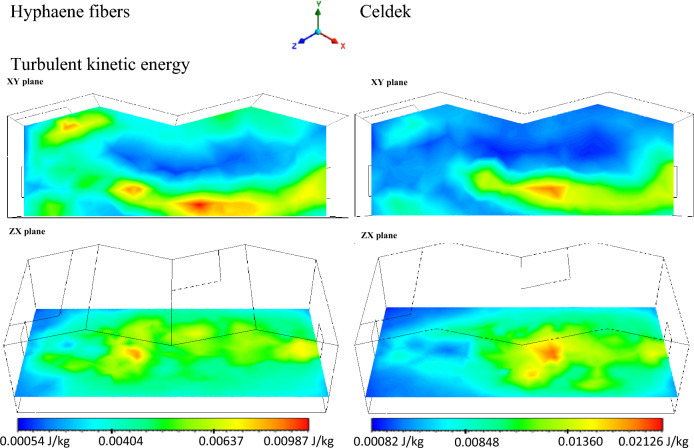
Turbulent kinetic energy contours.

**Figure 10:**
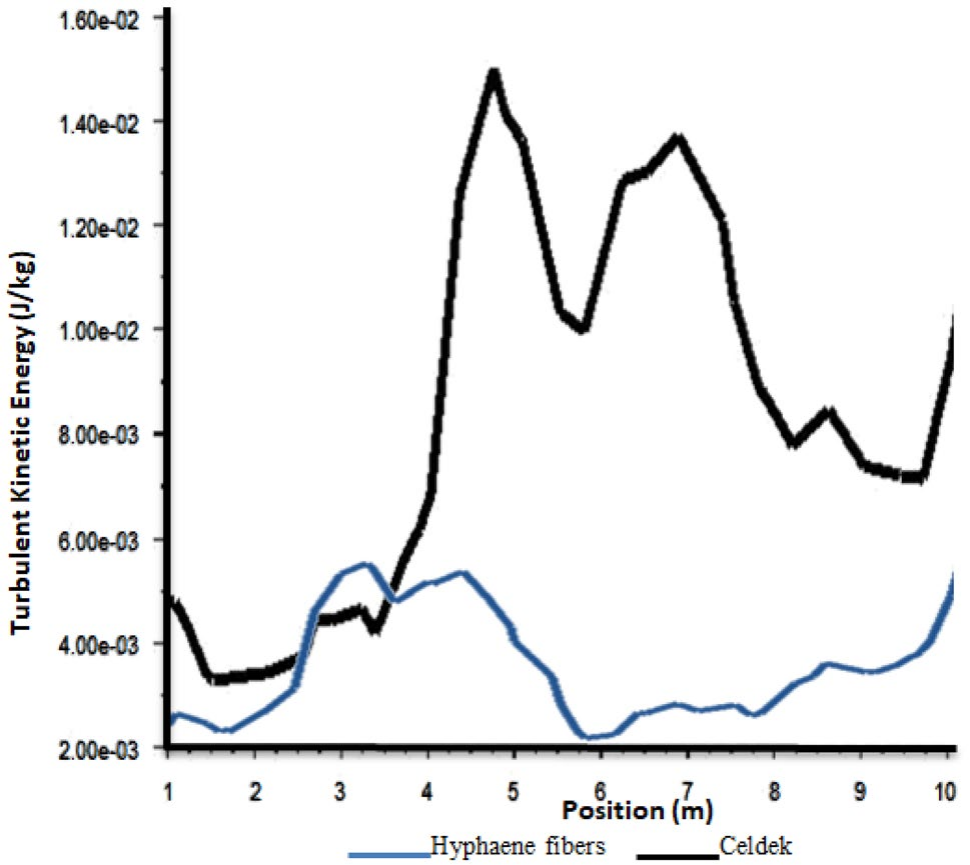
Plot of turbulent kinetic energy from inlet to outlet.

The turbulent kinetic energy (TKE) investigations are mostly conducted for greenhouse solar dryers compared to cooling system. The TKE representation in the evaporative cooling greenhouse, on its horizontal and vertical plans, gave important results that can help capture temperature distribution and air homogeneity in the greenhouse. For the two cooling pads, it was observed an important TKE increase far from the wet-pads. With difference to greenhouse solar dryers, cooling greenhouse TKE contours (Fig. 9) showed higher turbulence from 2 m (0.00404–0.00987 J/kg) and 4 m (0.00848–0.02126 J/kg) away from the cooling source or the inlet for Hyphaene fibers and Celdek pads respectively (fiGure 10). The observed TKE increase is linked to homogeneity of air temperature in the greenhouse at these distances. The experimental measured air velocity and temperature distributions in the greenhouse support these findings, where the maximum temperature was reached at 4 m for the two pads. It was also noticed from the CFD analysis plots that along the cooling greenhouse walls and near to the roof, the observed TKE values were weak, showing a global air inhomogeneity like for greenhouse solar dryers^21,25^. Therefore, additional fans could help to increase cooling air homogenization in the whole greenhouse volume^26^.

**Figure 11:**
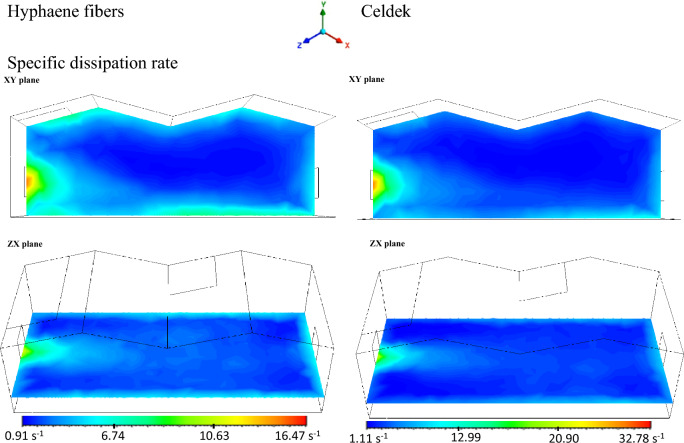
Specific dissipation rate contours.

The rate at which turbulent kinetic energy is converted into thermal internal energy per unit volume and time is known as the specific dissipation rate. Both contours showed higher dissipation rate at the inlet up to 2 m away from the cooling source (Fig. 11). A value of 32.78 s^−1^ was observed from Celdek pad air stream due to its higher velocity against 16.47 s^−1^ obtained from Hyphaene fibers pad.

## Conclusion

In this study, an eco-friendly and cost effective cooling solution was sought to replace conventional cooler in emerging cooling greenhouse technology under climate variability and change. To conduct this research work, the thermal properties and cooling performances of many biomass materials, locally used to cool water in remote areas, were preliminary investigated. The thermally most promising biomaterial, *Hyphaene thebaica* fiber, was used to design a new alternative pad (HF-pad). To improve the evaporative cooling greenhouse sustainability, it was powered by photovoltaic energy source. The preliminary simulation and field experimental data were used to assess the HF-pad and the evaporative cooling greenhouse performances against one of the largely used conventional pads (Celdek pad). The results showed that the alternative HF-pad microclimate parameters and cooling performances compete with the commercial C-pad. Indeed, the spread air from HF-pad was able to maintain the greenhouse temperature around 25 °C, bearable for most horticulture crops. The relative humidity in the greenhouse increased more than two times, and the maximum air moisture crossed 80%. The obtained high cooling performances (eff, cooling capacity, heat and mass transfers, COP) showed that HF-pad seemed thermally effective and 5 times cost effective than conventional pad. Computational fluid dynamic (CFD) analysis of the cooling air flow and the distribution of the key cooling parameters in the greenhouse showed that HF-pad produced cooler air compared to commercial C-pad. The CFD analysis also allowed to reach a better understanding of air flow and refrigerant air distribution in the greenhouse. The CFD analysis results correlate well with the experimental data and derived performances. Overall, HF-pad could be a valuable biomaterial candidate in evaporative cooling greenhouse.

## Data Availability

All data generated or analyzed in this study are included in this published article and its related optional files.
